# Quality by Design-Based Formulation Development of an Oral Semaglutide Tablet

**DOI:** 10.3390/pharmaceutics18040440

**Published:** 2026-04-01

**Authors:** Ji-Hyeon Yoon, Do-Hyub Kim, Joo-Eun Kim

**Affiliations:** 1 Department of Biopharmaceutical Chemistry, Kookmin University, Seoul 02707, Republic of Korea; ouokk0217@kookmin.ac.kr (J.-H.Y.); hyufree@kookmin.ac.kr (D.-H.K.); 2 Department of Biopharmaceutical Chemistry, School of Applied Chemistry, Kookmin University, Seoul 02707, Republic of Korea; 3 Department of Pharmaceutical Engineering, Kookmin University, Seoul 02707, Republic of Korea

**Keywords:** design of experiments, oral formulation, oral semaglutide, peptide drug delivery, quality by design, sodium caprate, semaglutide, type 2 diabetes mellitus

## Abstract

**Background**: This study aimed to investigate, from a scientific and formulation perspective, an oral semaglutide tablet incorporating sodium caprate (C10) as an intestinal absorption enhancer and to optimize its formulation performance using a Quality by Design (QbD)-based approach. Semaglutide—a peptide-based therapeutic—provides effective glycemic control and weight reduction; however, its extremely low oral bioavailability has limited administration to subcutaneous injection. Although various attempts have been made to improve peptide absorption, achieving consistent delivery through oral routes remains a significant challenge due to enzymatic degradation and poor membrane permeability. **Methods**: To overcome these limitations, an absorption enhancer (sodium caprate) was incorporated to enhance oral absorption, and a Quality by Design (QbD)-based approach was applied to systematically guide formulation development. Following the definition of the Quality Target Product Profile and critical quality attributes, risk assessments (Preliminary Hazard Analysis and Failure Mode and Effects Analysis) were conducted to identify key formulation factors. A design of experiments approach was then employed to determine the optimal tablet composition. **Results**: Consequently, the resulting formulation met all predefined quality criteria, including hardness, disintegration, friability, and content uniformity. In addition, the in vitro dissolution profile demonstrated a release pattern comparable to that of the reference product, with similarity factor values of 74.4, 74.7, and 71.3 at pH 1.2, 4.0, and 6.8, respectively. **Conclusions**: These findings indicate that the formulation can achieve consistent and reproducible quality performance as an oral semaglutide dosage form. The QbD-based formulation design strategy presented in this study provides a robust and broadly applicable approach for developing oral delivery systems for peptide drugs, including semaglutide, and ultimately provides useful formulation insight for future peptide-based oral delivery research.

## 1. Introduction

Glucagon-like peptide-1 receptor agonists (GLP-1 RAs) have become increasingly important in managing type 2 diabetes and obesity [1,2]. However, most approved GLP-1 RAs are formulated for subcutaneous administration and require daily or weekly administration [1]. These injection-based regimens are frequently associated with pain, needle-related anxiety, and local injection-site reactions, which can significantly diminish long-term treatment adherence [3]. Real-world observational data show that approximately half of patients discontinue injectable GLP-1 therapy within 12 months of initiation, and treatment persistence declines to ~30% at 24 months [3]. Additionally, these injectable formulations generally require cold-chain storage, creating logistical and distribution challenges and further reducing patient convenience. Gastrointestinal adverse events are more commonly reported with injectable formulations than with oral formulations, and these reactions remain a major contributor to treatment discontinuation [3]. Collectively, these limitations highlight the urgent need for alternative formulation strategies that enhance patient adherence and administration convenience [4].

Peptide therapeutics exhibit high potency and target specificity, establishing them as promising candidates in pharmaceutical development [1]. Nevertheless, the oral delivery of peptide drugs remains particularly challenging [5]. Peptides are highly susceptible to enzymatic degradation within the gastrointestinal tract, and their relatively large molecular size significantly restricts permeation across the intestinal epithelium [6]. In addition to enzymatic degradation, orally administered peptides also face pH-dependent conformational or chemical instability, limited permeability across the intestinal membrane, and variable gastrointestinal transit and absorption kinetics. These multiple physiological hurdles collectively contribute to the extremely low and inconsistent oral bioavailability commonly observed for peptide therapeutics. Overcoming these absorption-related barriers has long remained a major bottleneck in developing peptide therapeutics. In general, peptide candidates considered more suitable for oral delivery tend to possess relatively favorable features such as a sufficiently wide therapeutic window, relative stability in the gastrointestinal environment, a long systemic half-life, and relatively low clearance [7]. Therefore, advancing oral delivery technologies is essential to address the inherent limitations of injectable formulations and enhance patient convenience [1].

**Figure 1:**
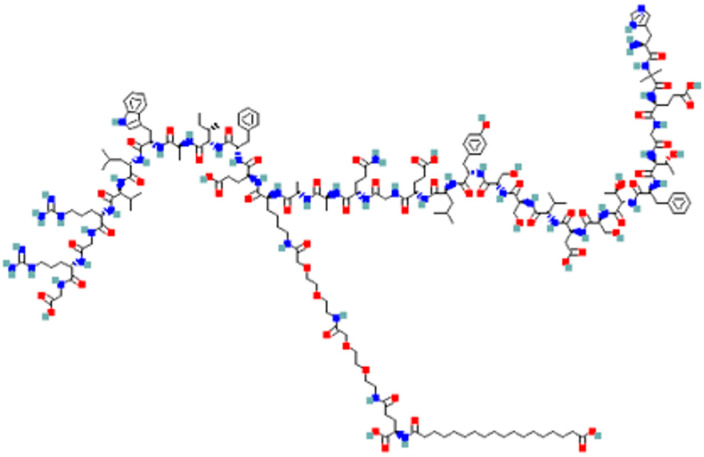
Chemical structure of the GLP-1 analog semaglutide (molecular weight: 4113.58 g/mol), obtained from PubChem (CID: 56843331). Abbreviation: GLP-1, glucagon-like peptide-1.

Semaglutide (molecular weight: 4113.58 g/mol) is a peptide-based GLP-1 analog engineered via strategic amino acid modifications to enhance resistance to dipeptidyl peptidase-4-mediated enzymatic degradation and extend its biological half-life [6]. The chemical structure of semaglutide is presented in Figure 1. It has been clinically validated for the treatment of diabetes and obesity and, following its initial approval as an injectable formulation, was subsequently commercialized as the first oral GLP-1 RA in the form of the SNAC-based product Rybelsus^®^, representing a major advance in oral peptide delivery [6]. The successful development of oral semaglutide requires formulation strategies capable of protecting the peptide from gastrointestinal degradation while promoting efficient intestinal absorption [4]. Owing to the harsh gastric environment and epithelial barriers, semaglutide—similar to other high-molecular-weight and highly hydrophilic peptides—exhibits limited permeability across epithelial tight junctions, ultimately resulting in low oral bioavailability [8]. Although a variety of formulation and delivery technologies have been investigated for oral peptide administration, including nanoparticle-based systems, self-emulsifying drug delivery systems, self-assembled carrier platforms, and peptide delivery approaches using permeation enhancers or enhancer-linked systems, many of these remain difficult to translate into commercially viable products because of formulation complexity, manufacturing challenges, scale-up limitations, reproducibility issues, and regulatory uncertainty [2,9]. In particular, nanoparticle- and complex DDS-based systems, while scientifically attractive, often present substantial practical barriers to robust product development and commercialization. From this perspective, permeation enhancer-based approaches remain among the most practical and translationally relevant formulation strategies for oral peptide delivery. Although oral semaglutide has already been successfully commercialized in the form of the SNAC-based product Rybelsus^®^, exploration of alternative absorption-enhancing strategies remains scientifically relevant for expanding formulation options in oral peptide delivery.

In this context, the present study was undertaken to investigate, from a scientific and formulation perspective, a sodium caprate (C10)-based oral semaglutide system as an alternative intestinal permeation strategy. Whereas SNAC primarily facilitates peptide absorption through localized modulation of the gastric microenvironment, C10 represents an alternative permeation enhancer that can influence epithelial permeability in the intestinal region. Given the larger absorptive surface area of the intestinal epithelium, investigation of intestinal permeation enhancers such as C10 may offer an additional scientifically relevant formulation strategy for oral peptide delivery. C10 is a widely studied non-proprietary permeation enhancer for oral peptide and protein delivery. C10 enhances intestinal permeation primarily through transiently modulating epithelial tight junctions to facilitate paracellular transport and through reversibly altering membrane phospholipid fluidity, thereby enabling limited transcellular uptake. Owing to its well-characterized, concentration-dependent permeation-enhancing mechanism, C10 is especially suitable for formulation optimization studies [8].

The concentration of C10 used in this study was selected based on previously reported oral peptide delivery systems employing comparable levels of absorption enhancers. Previous studies have demonstrated that approximately 300 mg of C10 can facilitate gastrointestinal absorption of peptide therapeutics [10]. Accordingly, a similar level of C10 was adopted in the present study to maintain consistency with previously reported enhancer-based oral peptide formulations.

Although relatively high levels of absorption enhancers can effectively improve gastrointestinal permeability of peptide drugs, their incorporation at high weight fractions may pose challenges for tablet manufacturability and dissolution consistency [8]. In the present study, the enhancer level was selected based on both previously reported literature evidence and preliminary in vivo pharmacokinetic evaluation in Sprague–Dawley rats. Therefore, the present study focused on optimizing the surrounding tablet matrix to accommodate C10, which is functionally effective but formulation-challenging. During formulation development, various factors, including the physicochemical properties of the active pharmaceutical ingredient (API), excipient compatibility, and process-related variables, may influence final product quality. In particular, under conditions where the absorption enhancer level is fixed, as in the present study, changes in other formulation components and their interactions may have a significant impact on tablet performance. Accordingly, a QbD approach was employed to systematically manage formulation-related variability and to enhance the reproducibility and scientific rigor of the development process [11].

An initial risk assessment (RA) was conducted to identify material attributes with potential impact on product quality, followed by the designation of critical material attributes (CMAs) [12,13]. The Quality Target Product Profile (QTPP) was prospectively defined prior to experimental formulation development and served as the primary design input within the QbD framework, ensuring that all subsequent activities were aligned with established target quality criteria [14,15].

Subsequently, a central composite design (CCD) within the design of experiments (DoE) framework was employed to optimize the formulation, overcoming the limitations of single-factor evaluation and enabling quantitative analysis of interaction effects between variables [16]. This systematic approach enabled predictive control of formulation performance and guided the development of a dosage form aligned with the predefined QTPP [11].

## 2. Materials and Methods

### 2.1. Materials

Semaglutide was obtained from Fugian Genohope Biotech Ltd. (Putian, China), Sinopep-Allsino Biopharmaceutical Co., Ltd. (Hangzhou, China), and Shanghai Wayx Biotechnology Co., Ltd. (Shanghai, China). Microcrystalline cellulose (MCC, Heweten 102) was purchased from JRS Pharma (Holzmühle 1, Rosenberg, Germany), and lactose monohydrate (Pharmatose 200M) was obtained from DFE Pharma (Goch, Germany). Povidone and crospovidone (Kollidon CL) were obtained from BASF (Ludwigshafen, Germany), and magnesium stearate was obtained from Faci Asia Pacific Pte. Ltd. (Merlimau PI, Jurong Island, Singapore). Sodium caprate (C10) was obtained from Tokyo Chemical Industry Co., Ltd. (Tokyo, Japan). Sodium *N*-[8-(2-hydroxybenzoyl)amino]caprylate (SNAC) was purchased from GLPBIO Inc. (Montclair, CA, USA). Acetonitrile and methanol (HPLC grade) were purchased from Duksan Co., Ltd. (Gyeonggi, Republic of Korea). Buffer solutions spanning pH 2.0–12.0 (extra-pure grade) were obtained from Deoksan Pharmaceutical Co., Ltd. (Ansan, Republic of Korea). Deionized water (18 MΩ·cm) was prepared in-house using a laboratory purification system. Analytical-grade reagents were employed throughout the experiments without further purification.

### 2.2. Physicochemical Properties of Active Pharmaceutical Ingredient

The solubility of semaglutide from various manufacturers was evaluated in deionized water, organic solvents—ethanol, methanol, and acetonitrile—and buffer solutions at pH 1.2, 2.0, 3.0, 4.0, 5.0, 6.8, 7.0, and 8.0. To comprehensively characterize the physicochemical behavior of the API, apparent and equilibrium solubility tests were conducted sequentially. For the apparent solubility assessment, ~10 mg of semaglutide was placed in each vial, followed by the addition of ~200 µL of solvent. The mixtures were gently stirred at 400 rpm until a visually clear solution formed, and the corresponding solvent-to-drug ratio was recorded. After confirming solution clarity, an excess amount of semaglutide was added to fresh vials, and the mixtures were continuously agitated at 400 rpm to achieve saturation. Samples (4 mL) were withdrawn at 2, 12, and 24 h, filtered through a 0.45 µm polytetrafluoroethylene (PTFE) membrane, and analyzed using high-performance liquid chromatography (HPLC) via an Agilent 1260 Infinity II system (Agilent Technologies, USA) under the chromatographic conditions described in Section 2.7.4 to determine the equilibrium solubility profile.

### 2.3. Compatibility Study Between Active Pharmaceutical Ingredient and Excipients

To identify excipients that do not induce physicochemical interactions with semaglutide, a compatibility study was conducted using an HPLC-based assay to monitor the API peak area. Each excipient was mixed with semaglutide at a 1:1 (*w*/*w*) ratio and placed in individual vials. The prepared samples were stored for 4 weeks in controlled chambers under two environmental conditions: 25 ± 2 °C/60 ± 5% RH (ambient) and 40 ± 2 °C/75 ± 5% RH (accelerated). At predetermined intervals, samples were collected to evaluate changes in appearance and assay content. The excipients evaluated included MCC, lactose, low-substituted hydroxypropyl cellulose, hydroxypropyl cellulose, talc, colloidal silicon dioxide, magnesium stearate, sodium starch glycolate, sodium stearyl fumarate, croscarmellose sodium, crospovidone, povidone, copovidone, polyethylene glycol, sodium lauryl sulfate, sodium hydrogen carbonate, sodium carbonate, magnesium carbonate, salicylic acid, sodium *N*-[8-(2-hydroxybenzoyl)amino]caprylate (SNAC), and C10. The samples were stored in transparent containers and evaluated immediately after blending, and after 2 and 4 weeks of storage. Following visual inspection, the semaglutide content was quantified using HPLC (Agilent 1260 Infinity II, Agilent Technologies, Santa Clara, CA, USA), following the assay procedure described in Section 2.7.4.

### 2.4. In Vivo Pharmacokinetic Evaluation of C10-Enabled Oral Semaglutide Formulations in Sprague–Dawley Rats

Male Sprague–Dawley rats (7 weeks old, 230–270 g) were purchased from Young Bio (Seongnam, Gyeonggi, Republic of Korea). Animals were housed under controlled laboratory conditions (temperature 21 ± 2 °C, relative humidity 35–65%, 12 h light/dark cycle, and 10–15 air changes per hour) and allowed free access to standard chow and water. All experimental procedures were approved by the Institutional Animal Care and Use Committee of Dongduk Women’s University (approval no. 202407-01, approved on 18 July 2024).

Before drug administration, animals were fasted for approximately 16 h while maintaining free access to water. The rats were randomly assigned to four treatment groups (*n* = 5 per group). The treatment groups consisted of (1) semaglutide (3 mg/kg); (2) semaglutide (3 mg/kg) with C10 (100 mg/kg); (3) semaglutide (3 mg/kg) with C10 (200 mg/kg); and (4) semaglutide (3 mg/kg) with SNAC (200 mg/kg). Semaglutide and the permeation enhancers were freshly dispersed in an aqueous vehicle prior to administration and delivered orally via gavage, with the dosing volume adjusted according to body weight.

Blood samples (~350 µL) were collected from the jugular vein into K_2_-EDTA tubes at predetermined time points (pre-dose, 0.5, 1, 2, 4, 6, 8, and 24 h post-dose). Plasma was obtained by centrifugation at 12,000 rpm for 5 min at 4 °C. The separated plasma samples were transferred to labeled tubes and stored at −70 °C until analysis. Plasma semaglutide concentrations were quantified using liquid chromatography–tandem mass spectrometry (LC–MS/MS; QTRAP^®^ 6500, AB Sciex LLC, Framingham, MA, USA).

### 2.5. Quality Target Product Profile, Critical Quality Attribute, and Risk Assessment of Critical Material Attribute (Preliminary Hazard Analysis, Failure Mode and Effects Analysis)

Formulation development was conducted under a QbD framework [17]. A QTPP was first established to ensure patient safety and convenience [11]. The QTPP defined the intended route of administration, dosage form, strength, and key quality attributes, specifying the desired characteristics of the final product, as summarized in Table 1.

Based on the QTPP, several quality attributes relevant to product performance were identified, including tablet hardness, disintegration time, friability, content uniformity, and dissolution [13], as summarized in Table 2. Among these, dissolution was designated as the primary CQA for formulation optimization, as it directly reflects in vitro equivalence to the reference product and serves as a key determinant in generic drug development [11]. The remaining attributes were evaluated as supporting quality attributes to confirm acceptable tablet quality and manufacturing robustness. Tablet hardness was monitored as a supporting mechanical attribute to ensure adequate tablet integrity and manufacturability during formulation development.

A two-step risk assessment strategy was subsequently implemented to systematically evaluate the influence of material attributes (MAs) on CQAs [18]. In the first stage, a Preliminary Hazard Analysis (PHA) was conducted to classify MAs into high-, medium-, or low-impact. High-impact MAs were then further analyzed using Failure Mode and Effects Analysis (FMEA) [19]. Each MA was scored for severity, occurrence, and detectability on a 1–5 scale, and the Risk Priority Number (RPN) was calculated as the product of these scores [20]. MAs with an RPN ≥ 30 were classified as CMAs and were subsequently used as input parameters for formulation optimization. A CCD was employed using Minitab software (version 22; Minitab^®^, LLC, State College, PA, USA) to explore the design space and establish optimal formulation conditions [21].

### 2.6. Formulation Studies of Semaglutide Tablets

#### 2.6.1. Composition of Semaglutide Tablets

Prior to the QbD-based formulation optimization, a preliminary excipient screening study was conducted to evaluate the compatibility of semaglutide with candidate excipients and to assess their influence on formulation feasibility. Several commonly used tablet excipients representing different functional roles in the tablet matrix (e.g., fillers, binders, and disintegrants) were investigated. Based on these results, excipients demonstrating acceptable compatibility and formulation feasibility were selected for subsequent risk assessment and DoE optimization. The quantitative composition of non-CMA excipients was fixed based on these screening outcomes and commonly accepted pharmaceutical formulation ranges.

The oral semaglutide formulation was developed through systematically selecting and optimizing excipients that preserved the drug assay and ensured chemical stability upon combination with the active pharmaceutical ingredient, based on the predefined QTPP, CQA assessment, and excipient compatibility studies. Furthermore, to address concerns related to peptide stability and to ensure adequate content uniformity, tablets were manufactured using a dry granulation process. The qualitative and quantitative composition of the optimized semaglutide monolayer tablet formulation is summarized in Table 3. The formulation consisted of semaglutide (14 mg), lactose (300 mg), sodium caprate (300 mg), croscarmellose sodium (17 mg), copovidone (13 mg), and magnesium stearate (8 mg), resulting in a total tablet weight of approximately 652 mg.

#### 2.6.2. Manufacturing Process of Semaglutide Tablets

Semaglutide (14 mg), lactose (300 mg), sodium caprate (300 mg), croscarmellose sodium (17 mg), and copovidone (13 mg) were accurately weighed and manually blended for approximately 100 mixing cycles to ensure uniform distribution. The blended mixture was then subjected to dry granulation. Slugging was performed using a rotary tablet press (PR-LM 08, PTK Co., Ltd., Gimpo, Republic of Korea) equipped with a 15.0 mm round punch and die set under a compression force of 21 kN. The resulting slugs were milled through a 3.0 mm sieve. Magnesium stearate (8 mg) was subsequently added as a lubricant and mixed with the granules. Finally, the granules were compressed into tablets using the same rotary press equipped with a 16.7 × 8.3 mm oblong punch and die set under a compression force of 4.52 kN.

### 2.7. Characteristics Evaluation of Semaglutide Tablets

#### 2.7.1. Hardness

Tablet hardness influences critical dosage form properties, including drug release behavior, disintegration rate, and resistance to mechanical stress, and is therefore regarded as a key quality attribute for assessing the risk of tablet breakage during handling, distribution, and storage. In this study, hardness testing was conducted to evaluate the physical strength and mechanical integrity of the formulation. The hardness of the semaglutide single-layer tablets was measured using a digital hardness tester (model YD-II, LABOAO, Zhengzhou, China). Measurements were obtained from six tablets, and results were reported in kilopond (kp). Based on predefined development criteria, an acceptable hardness range of 7–9 kp was established.

#### 2.7.2. Disintegration

Tablet disintegration time is a CQA that directly influences the drug release rate during dissolution behavior. In this study, disintegration testing was evaluated according to the Korean Pharmacopoeia guidelines [16]. Purified water served as the disintegration medium, and the test was conducted using a BJ-2 disintegration tester (Nanbei Instrument Ltd., Zhengzhou, China). Six tablets were individually placed in separate glass tubes, and the water bath temperature was maintained at 37 ± 0.5 °C. Each tablet was observed until complete disintegration, and compliance with the specified time limit was recorded. Disintegration time and inter-tablet uniformity were subsequently compared and analyzed.

#### 2.7.3. Friability

Tablet friability is a key indicator of the resistance of finished pharmaceutical products to mechanical stress encountered during manufacturing, handling, and distribution. Friability testing is used to predict the propensity for tablet chipping or abrasion, thereby ensuring physical integrity and minimizing the risk of quality defects during storage and patient use. In this study, tablet friability of the semaglutide single-layer formulation was evaluated using a CS-4 friability tester (Minhua Pharmaceutical Machinery Co., Ltd., Shanghai, China) following the Korean Pharmacopoeia (KP) and United States Pharmacopeia (USP) <1216> guidelines [21]. In total, 10 tablets were placed in the test drum and rotated 100 times at 25 rpm. Friability (%) was calculated from the difference in total tablet weight before (W1) and after (W2) testing. A weight loss ≤ 1.0% was considered acceptable.

$$Friability\left(\%\right)=\frac{W_{1}-W_{2}}{W_{1}}\times 100(\%)$$

#### 2.7.4. Assay (High-Performance Liquid Chromatography)

The semaglutide content was quantified using an HPLC system (Agilent 1260 Infinity II, Agilent Technologies, Santa Clara, CA, USA) equipped with a UV–Vis detector. Chromatographic separation was performed using an Agilent C18 column (4.6 × 150 mm, 5 µm). The column temperature was maintained at 30 °C, and the detection wavelength was set at 230 nm. The flow rate was 0.9 mL/min, and the injection volume was 35 µL. Methanol was used as the sample diluent. The mobile phase consisted of (A) 0.08% trifluoroacetic acid in deionized water and (B) 0.08% trifluoroacetic acid in acetonitrile. Gradient elution was applied as follows: 0–18.0 min, 85% A/15% B; 18.0–18.1 min, 15% A/85% B; and 18.1–22.0 min, 85% A/15% B. The analytical method was validated in terms of linearity, precision, accuracy, and reproducibility for the quantitative determination of semaglutide.

#### 2.7.5. Content Uniformity

Content uniformity evaluation is critical for ensuring consistent dosing across individual tablets, thereby supporting patient safety and reproducible therapeutic performance. Testing was conducted according to the USP <905> Uniformity of Dosage Units guideline using ten semaglutide monolayer tablets [22]. Each tablet was completely dissolved in an appropriate solvent, and semaglutide content was quantified using HPLC. Compliance was confirmed by demonstrating that the content of each unit fell within ±15% of the mean value.

#### 2.7.6. In Vitro Dissolution

The dissolution performance of the semaglutide monolayer tablets was compared with that of the reference product using a USP-compliant dissolution apparatus (708-DS, Agilent, Seoul, Republic of Korea). Rybelsus^®^ 14 mg tablets served as the reference product. Dissolution testing was conducted according to USP <711> using Apparatus II (paddle method) at a paddle rotation speed of 50 rpm in 900 mL of dissolution medium maintained at 37 ± 0.5 °C [23]. For the study, media at pH 1.2 and 4.0 contained 0.75% (*w*/*v*) Brij^®^ 35, while the pH 6.8 medium, without surfactant, served as the comparative dissolution medium. The surfactant-containing dissolution medium was specifically selected to maintain sink conditions and to minimize solubility-limited artifacts, thereby allowing a clearer assessment of formulation-related effects on drug release. This approach was intended for comparative formulation assessment rather than for direct simulation of physiological gastric conditions. Dissolution testing was performed following MFDS dissolution guidelines [23]. At predetermined time points (5, 10, 15, 20, 30, 45, and 60 min), 4 mL aliquots were withdrawn from each of the 12 vessels. The obtained samples were subsequently filtered through 0.45 µm cellulose acetate membranes for pH 1.2 and 4.0 media, and through 0.45 µm PTFE membranes for the pH 6.8 medium. Quantitative analysis of the filtrates was conducted using HPLC via an Agilent 1260 Infinity II system (Agilent Technologies, Santa Clara, CA, USA). The similarity factor (f_2_) was calculated from the resulting dissolution profiles to assess equivalence with the reference product in accordance with regulatory guidelines [23].

## 3. Results and Discussion

### 3.1. Physicochemical Properties of the Active Pharmaceutical Ingredient

Three semaglutide API sources (Genohope, Sinopep, and WYX) were evaluated for solubility across various solvents and pH conditions as a foundational step in formulation development. Solubility is a critical physicochemical parameter that directly influences in vivo absorption; therefore, accurate characterization is essential for rational formulation design.

**Figure 2:**
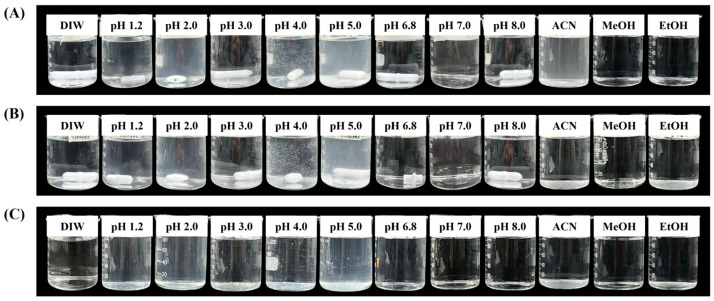
Changes in the appearance of semaglutide from (**A**) Genohope, (**B**) Sinopep, and (**C**) WYX in various solvents (apparent solubility test).

To characterize semaglutide dissolution properties, an apparent solubility test was first conducted. All experiments were conducted under identical magnetic stirring conditions, and the photographs were taken after completion of the solubility tests. Representative changes in the appearance of semaglutide from different API sources across various solvents and pH conditions are shown in Figure 2. All three API sources exhibited low solubility in the pH 1.2–5.0 range and were classified as “practically insoluble” according to USP and KP standards [24]. Conversely, in distilled water and under neutral to alkaline conditions (pH ≥ 6.8), all three APIs were classified as “freely soluble,” indicating a pronounced solubility shift between gastric and intestinal environments. In organic solvents, all APIs showed high solubility in methanol (freely soluble), “very slightly soluble” profile in ethanol, and remained insoluble in acetonitrile. The solubility of semaglutide in organic solvents was evaluated to support analytical sample preparation and method development.

**Figure 3:**
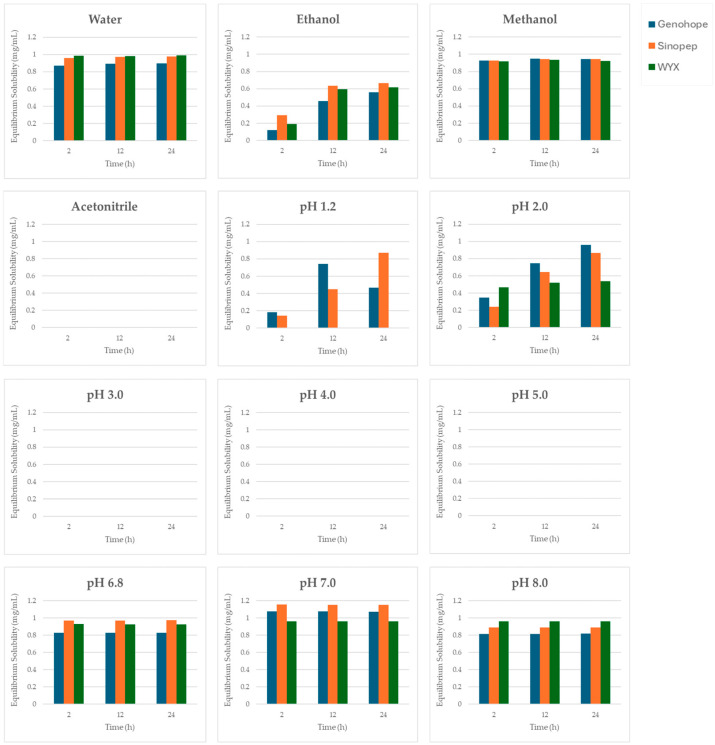
Equilibrium solubility profiles of semaglutide obtained from three API sources (Genohope, Sinopep, and WYX) in various solvents at different time points (2, 12, and 24 h).

Subsequently, an equilibrium solubility test was performed at 2, 12, and 24 h, and the equilibrium solubility profiles of the three API sources across different solvents are presented in Figure 3. Differences among API sources became more pronounced under acidic conditions. At pH 1.2, Sinopep remained “sparingly soluble,” indicating relatively higher solubility than the other sources, while Genohope was classified as “very slightly soluble,” representing the primary distinction between these two APIs. At pH 2.0, both APIs fell within the “sparingly soluble” category, with Sinopep exhibiting slightly higher solubility. From pH 3.0–5.0, both sources were insoluble, while at pH ≥ 6.8, they consistently exhibited freely soluble behavior. Similar trends were observed in organic solvents, with both APIs freely soluble in methanol, very slightly soluble in ethanol, and insoluble in acetonitrile.

The WYX API exhibited the least favorable solubility profile under acidic conditions. At pH 1.2, it remained completely insoluble, and at pH 2.0, it was classified as very slightly soluble, indicating significantly lower solubility than Sinopep and Genohope. Consistent with the other two sources, WYX transitioned freely soluble at pH ≥ 6.8 and exhibited comparable solubility in methanol and ethanol. Therefore, the most distinguishing characteristic of the WYX source is the markedly reduced solubility under acidic conditions.

Overall, solubility differences among the three semaglutide APIs were most pronounced at pH 1.2 and 2.0, establishing the ranking as Sinopep > Genohope > WYX. Conversely, under neutral and alkaline conditions (pH 6.8–8.0) and in methanol, solubility was comparable across all sources, resulting in reduced differentiation among manufacturers. These findings indicate that dissolution behavior under acidic conditions represents a critical determinant for early-stage formulation screening, particularly for oral dosage forms involving gastric transit and initial drug release.

Under the formulation conditions applied in this study, each tablet contained 14 mg of semaglutide, and dissolution testing was conducted in 900 mL of dissolution medium, corresponding to a theoretical concentration of ~0.016 mg/mL. According to regulatory guidelines, sink conditions are considered satisfied when the saturation solubility is ≥3-fold the theoretical concentration (≥0.048 mg/mL).

Results from the 24 h equilibrium solubility test demonstrated that all three API sources exceeded this sink criterion. Moreover, even the API source exhibiting the lowest solubility remained within at least the “very slightly soluble” category according to the KP and USP criteria. These findings suggest that semaglutide possesses sufficient solubility capacity under the dissolution media conditions employed in this study.

Particularly, given the inherently low intrinsic solubility of semaglutide under acidic conditions, dissolution media were supplemented with a surfactant to maintain sink conditions at low pH. This approach enabled objective evaluation of the intrinsic release behavior of the drug without artificial constraints imposed by solubility limitations.

In summary, sink conditions were confirmed for all API sources. The solubility data obtained at pH 1.2–2.0 provided valuable baseline information for understanding the initial gastric release characteristics of oral semaglutide formulations and for guiding optimal formulation design.

### 3.2. Compatibility Study Between Active Pharmaceutical Ingredient and Excipients

The standard practice for evaluating excipient compatibility in pharmaceutical formulations involves assessing the formation of degradation products using the HPLC-related substance test [25]. This approach provides a comprehensive assessment of chemical stability and drug safety by enabling the detection and quantification of impurities and API-derived degradation products. However, this study was limited by the absence of an officially validated related substance method for semaglutide.

In accordance with ICH Q1A (R2) and Q6A guidelines, which recognize that a reduction in API assay value directly reflects chemical instability, the HPLC content assay (assay) was employed as a primary screening tool. This method was used to preliminarily detect potential incompatibilities between semaglutide and excipients [26], allowing rapid detection of excipients with a high risk of interaction.

**Figure 4:**
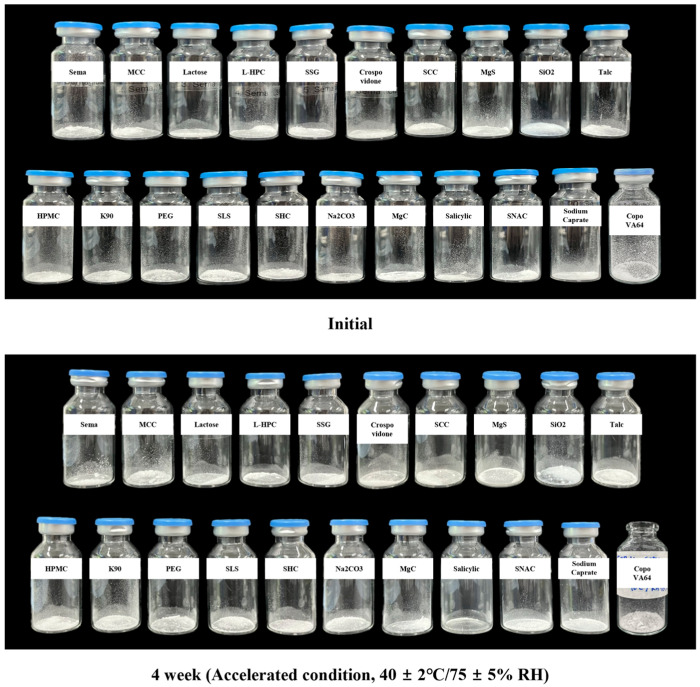
Visual assessment of compatibility between semaglutide and excipients, showing the absence of observable physical changes after storage.

For compatibility screening, semaglutide was blended with each excipient at a 1:1 (*w*/*w*) ratio and stored for 4 weeks under two stability conditions: ambient (25 ± 2 °C/60 ± 5% RH) and accelerated (40 ± 2 °C/75 ± 5% RH). In most combinations, semaglutide assay values remained within 95–105% of the initial content, indicating no significant compatibility concerns, as summarized in Table 4. Visual inspection confirmed the absence of macroscopic physical changes, such as discoloration or aggregation, across the evaluated mixtures. The representative visual appearance of the semaglutide–excipient mixtures under initial and accelerated storage conditions is documented in Figure 4.

However, semaglutide assay values remained stable at the initial and 2-week time points when combined with sodium lauryl sulfate (SLS), but declined sharply to ~0.2% after 4 weeks under both storage conditions, indicating near-complete degradation. This pronounced instability is potentially attributable to strong electrostatic and hydrophobic interactions between the anionic surfactant and peptide chain. SLS can penetrate the hydrophobic core of peptides and disrupt the hydrogen-bonding network, inducing irreversible denaturation and secondary/tertiary structural collapse, thereby accelerating chemical degradation [27]. Similarly, when combined with salicylic acid, semaglutide was undetectable after storage, indicating extensive degradation in acidic excipient environments. Given the susceptibility of peptides to acid-catalyzed degradation, these observations are consistent with the general tendency of peptide-based drugs to exhibit chemical instability under acidic conditions.

Overall, with the exception of SLS and salicylic acid, most excipients demonstrated acceptable preliminary compatibility with semaglutide. Nevertheless, the absence of related substances profiling is acknowledged as a limitation of this study, as assay retention alone cannot exclude the formation of potentially harmful degradation products or low-level degradants specific to peptide instability. In the absence of a validated related substance method at the time of this study, assay-based monitoring was employed as a pragmatic screening approach to detect substantial API loss during compatibility evaluation.

The excipient screening indicated that the selected tablet excipients showed acceptable compatibility with semaglutide and did not adversely affect the initial dissolution performance. Based on these findings, this combination of excipients was selected for subsequent risk assessment and DoE-based formulation optimization. Despite this limitation, the findings provide valuable baseline data for excipient screening and prioritization and underscore the need to establish a dedicated related substances method for semaglutide. Future studies will include the development and validation of a dedicated stability-indicating related substance method to enable comprehensive degradant characterization and to further strengthen excipient selection criteria.

### 3.3. Pharmacokinetic Evaluation of Oral Semaglutide in Sprague–Dawley Rats

**Figure 5:**
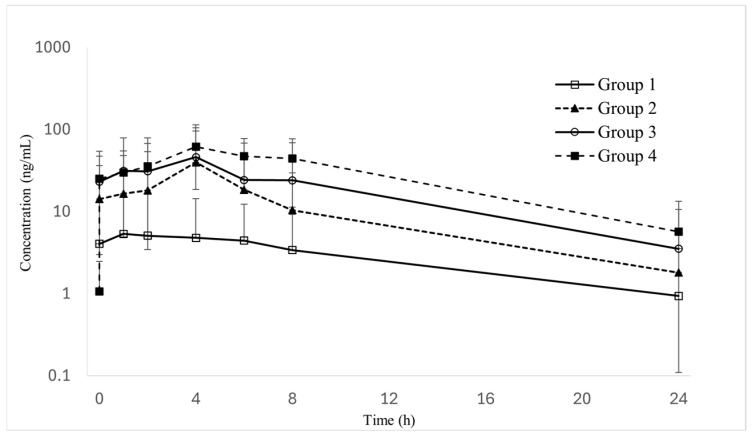
Plasma concentration–time profiles of semaglutide in rats following oral administration of C10- or SNAC-based formulations (*n* = 5).

Oral administration of semaglutide in Sprague–Dawley rats demonstrated a clear dose-dependent increase in systemic exposure when the permeation enhancer C10 was incorporated into the formulation. The plasma concentration–time profiles are shown in Figure 5, and the corresponding pharmacokinetic parameters are summarized in Table 5.

Oral administration of semaglutide alone resulted in minimal systemic exposure, with a Cmax of 9.9 ng/mL and an AUClast of 58.9 ng·h/mL. The addition of C10 significantly enhanced semaglutide absorption. When C10 was administered at 100 mg/kg, systemic exposure increased markedly, yielding an AUClast of 230.5 ng·h/mL. Further increasing the C10 dose to 200 mg/kg resulted in additional enhancement of systemic exposure (AUClast 346.9 ng·h/mL), indicating a dose-dependent increase in semaglutide absorption with increasing C10 concentration.

To compare the absorption-enhancing efficiency of C10 with the clinically used enhancer SNAC, systemic exposure was evaluated at an equivalent enhancer dose of 200 mg/kg. Under these conditions, C10 (200 mg/kg) and SNAC (200 mg/kg) produced comparable systemic exposure, with AUClast values of 346.9 ng·h/mL and 503.1 ng·h/mL, respectively.

Following peak concentration, all formulations exhibited a mono-exponential elimination phase, and no formulation-related adverse clinical signs were observed during the study period. Collectively, these findings demonstrate that C10 enhances the oral absorption of semaglutide in a dose-dependent manner, and that C10 at 200 mg/kg provides systemic exposure comparable to that achieved with SNAC at the same dose.

### 3.4. Quality Target Product Profile, Critical Quality Attributes, and Risk Assessment of Critical Material Attributes Using Preliminary Hazard Analysis and Failure Mode and Effects Analysis

The QTPP for the oral semaglutide formulation was established, and the corresponding CQAs were defined accordingly. The QTPP included the following key product attributes: indication, route of administration, dosage form, dosage strength, appearance, identification, assay, content uniformity, and dissolution performance. Based on these predefined targets, the key CQAs associated with formulation performance and in vitro equivalence were identified as content uniformity, dissolution behavior (initial release and dissolution rate up to 30 min at pH 1.2, 4.0, and 6.8), disintegration, and friability. These attributes were designated as critical indicators to ensure formulation safety and efficacy.

To proactively identify variables that may influence the CQAs, a PHA was conducted. A broad range of potential risk factors was evaluated, including API characteristics, absorption enhancer properties, and excipient levels such as filler, binder, disintegrant, and lubricant. Among these factors, variables associated with low formulation-related risk or fixed based on prior biological evaluation were excluded from further assessment. While the absorption enhancer content was fixed during formulation optimization based on prior biological evaluation, it was intentionally included in the RA to detect potential formulation-related risks associated with deviations from the predefined level. Subsequently, FMEA was applied to the shortlisted variables. Severity was defined as the potential effect on CQAs, occurrence as the likelihood of variation during processing, and detectability as the ability to identify such variation during routine quality evaluation. Each parameter was scored on a 1–5 scale (1 = low risk, 5 = high risk), and the RPN was calculated as the product of these three parameters to enable quantitative prioritization. The results of the PHA and FMEA are summarized in Table 6.

RA revealed that filler and binder levels exerted direct and indirect effects on dissolution performance and tablet mechanical strength and exhibited relatively higher RPN scores. Consequently, these two variables were designated as the final CMAs. Conversely, lubricants and other excipients exhibited low association with most CQAs, received low FMEA scores and were, therefore, excluded from CMA selection. Within the tested range, filler level had minimal effect on key quality attributes, indicating that it primarily functions as a robustness factor rather than a performance-driving variable.

Based on the selected CMAs, the amounts of filler (X1) and binder (X2) were designated as critical formulation variables and target operating ranges were defined. A DoE approach using a CCD was applied to systematically investigate their effects on CQAs in accordance with QbD-based formulation optimization recommendations. The experimental design is summarized in Table 7. The CCD was selected for its ability to evaluate nonlinear effects and potential interaction terms, making it suitable for defining a formulation design space. Dissolution at pH 1.2, 4.0, and 6.8 (10, 15, and 30 min) and friability were designated as response variables. The actual quantitative ranges corresponding to the coded factor levels were as follows: the filler level (X_1_), representing lactose, ranged from 50 to 300 mg, while the binder level (X_2_), corresponding to copovidone, ranged from 0 to 46 mg.

DoE analysis demonstrated a significant F-value (*p* < 0.05) in analysis of variance (ANOVA), with both the coefficient of determination (R^2^) and adjusted R^2^ > 0.9, indicating strong model predictability. The relationship between the formulation variables and the response was described using a quadratic regression model as follows:

$$Y=\beta_{0}+\beta_{1}X_{1}+\beta_{2}X_{2}+\beta_{12}X_{1}X_{2}+\beta_{11}X_{1}^{2}+\beta_{22}X_{2}^{2}$$

where $X_{1}$ represents the filler level (lactose) and $X_{2}$ represents the binder level (copovidone). Furthermore, the lack-of-fit test was not significant (*p* > 0.05), confirming model adequacy. Residual diagnostic analysis also satisfied assumptions of normality, homoscedasticity, and independence assumptions, thereby validating the statistical model. All statistical analyses and DoE modeling were performed using Minitab statistical software (version 22; Minitab^®^, LLC, State College, PA, USA). The experimental run order was randomized using the randomization function within the Minitab software to minimize potential systematic bias.

Main effects analysis revealed that binder level consistently and significantly influenced dissolution behavior and mechanical properties. Increasing binder concentrations delayed tablet disintegration and slowed dissolution while reducing friability <1.5%, thereby enhancing tablet robustness. Conversely, within the tested range, filler level had minimal effect on key quality attributes and primarily served as a robustness factor. These findings indicate that the formulation exhibits high tolerance to minor fluctuations in filler concentration, ensuring consistent product quality and providing advantages for manufacturing flexibility and process reliability during potential scale-up.

**Figure 6:**
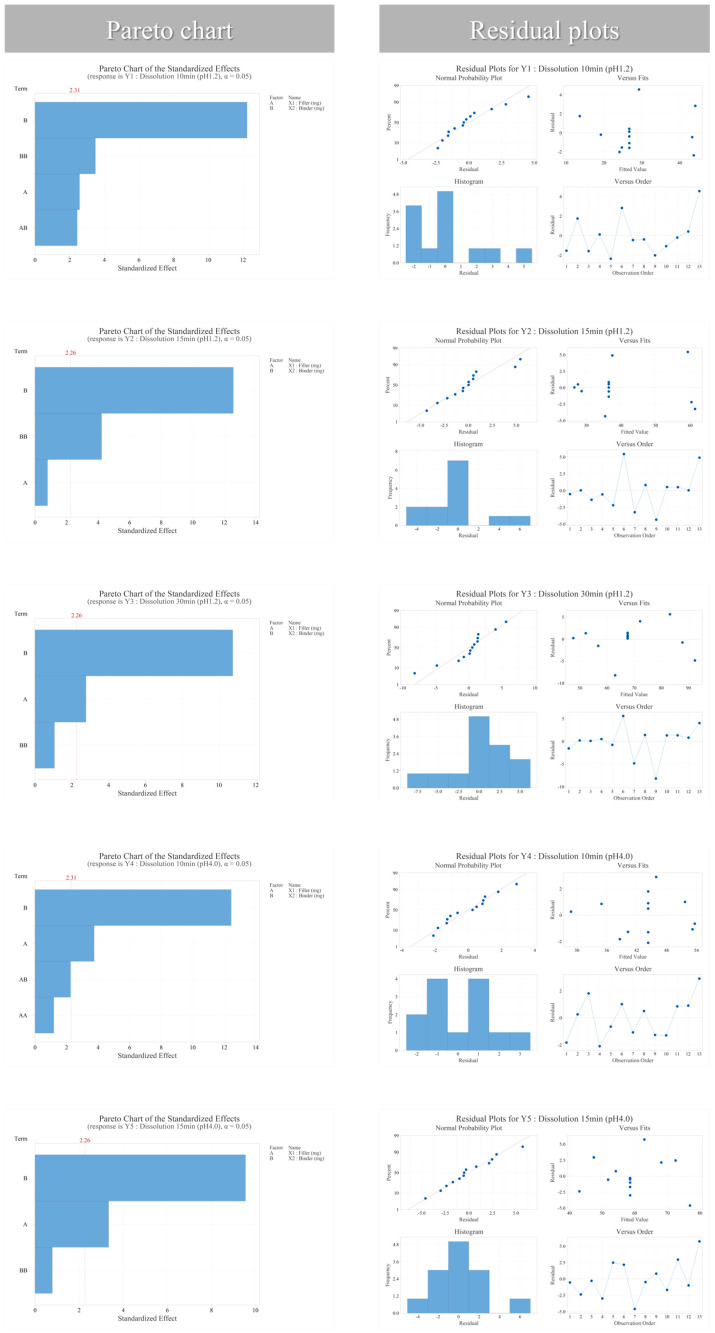

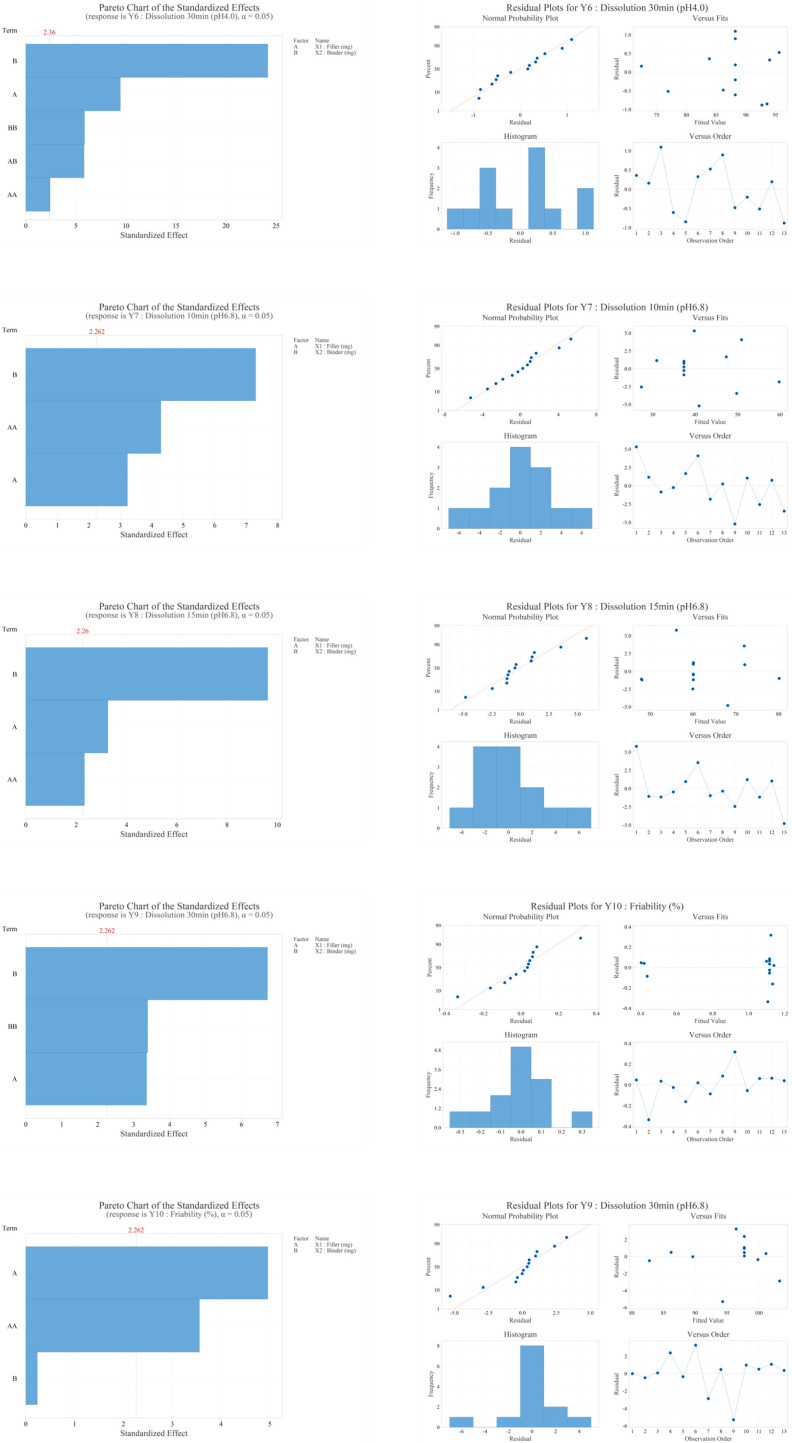

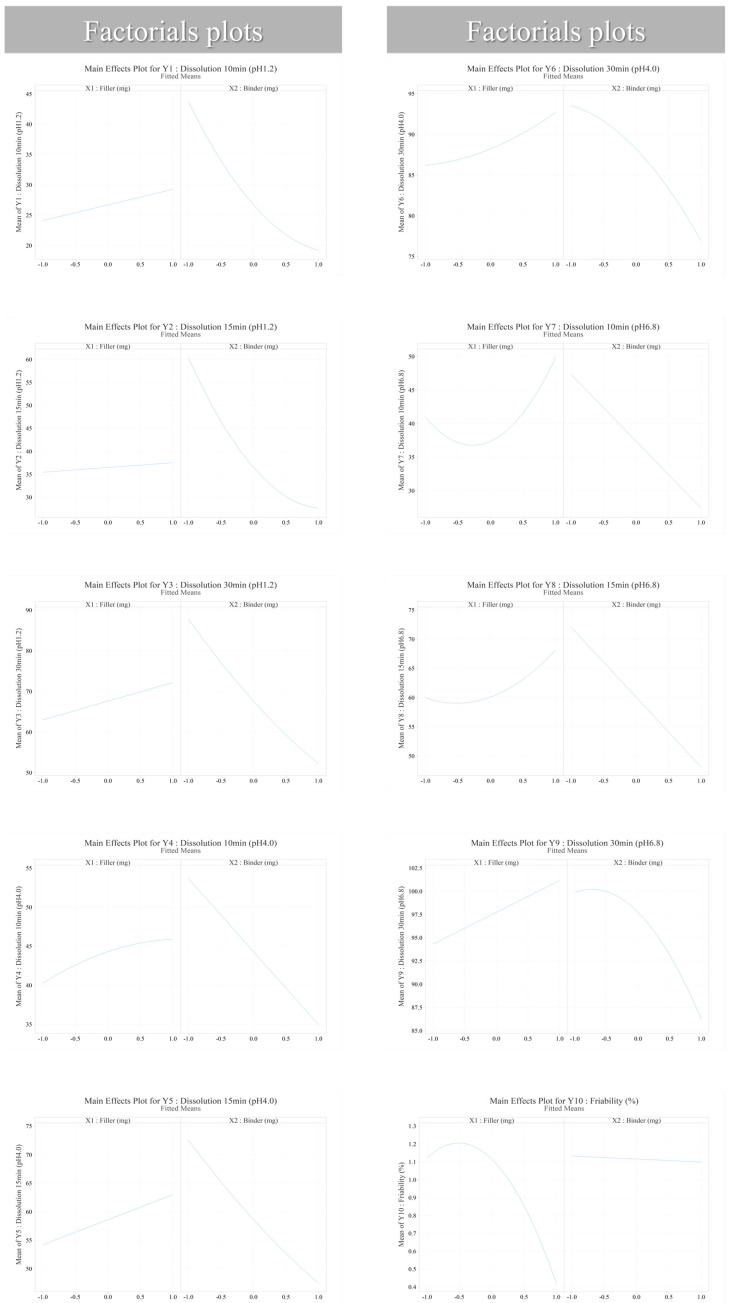

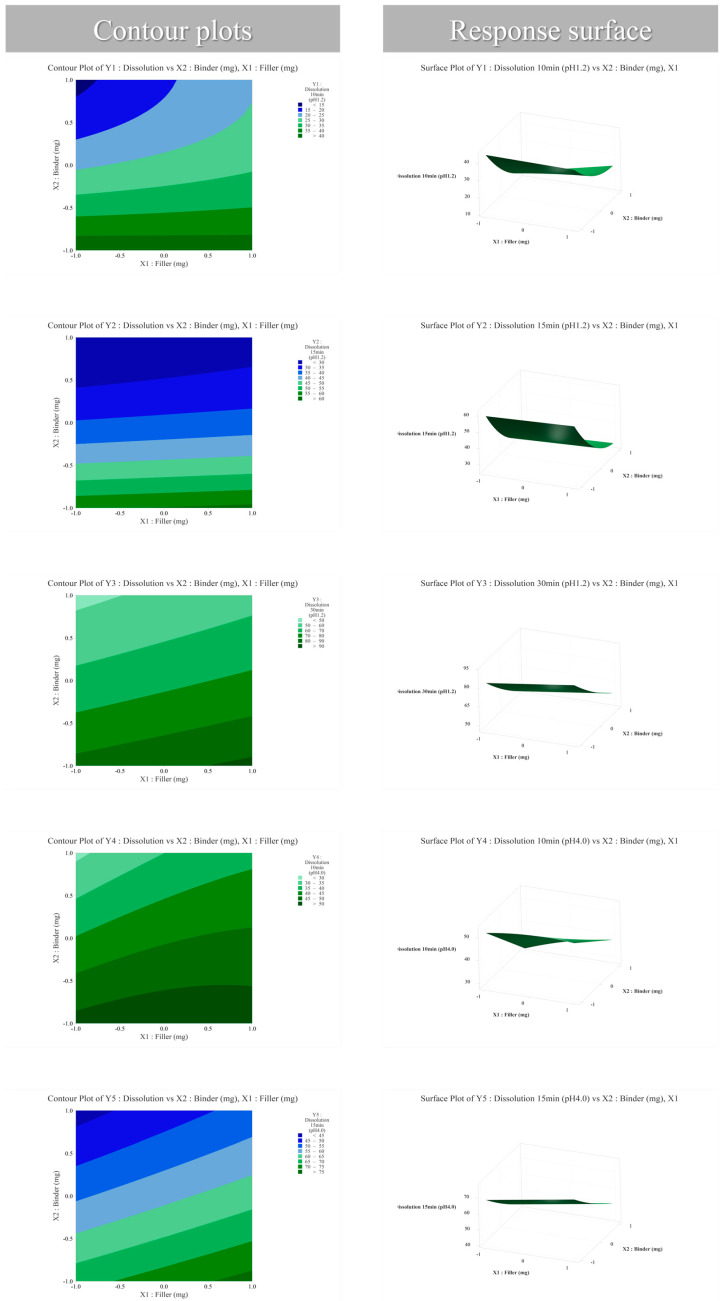

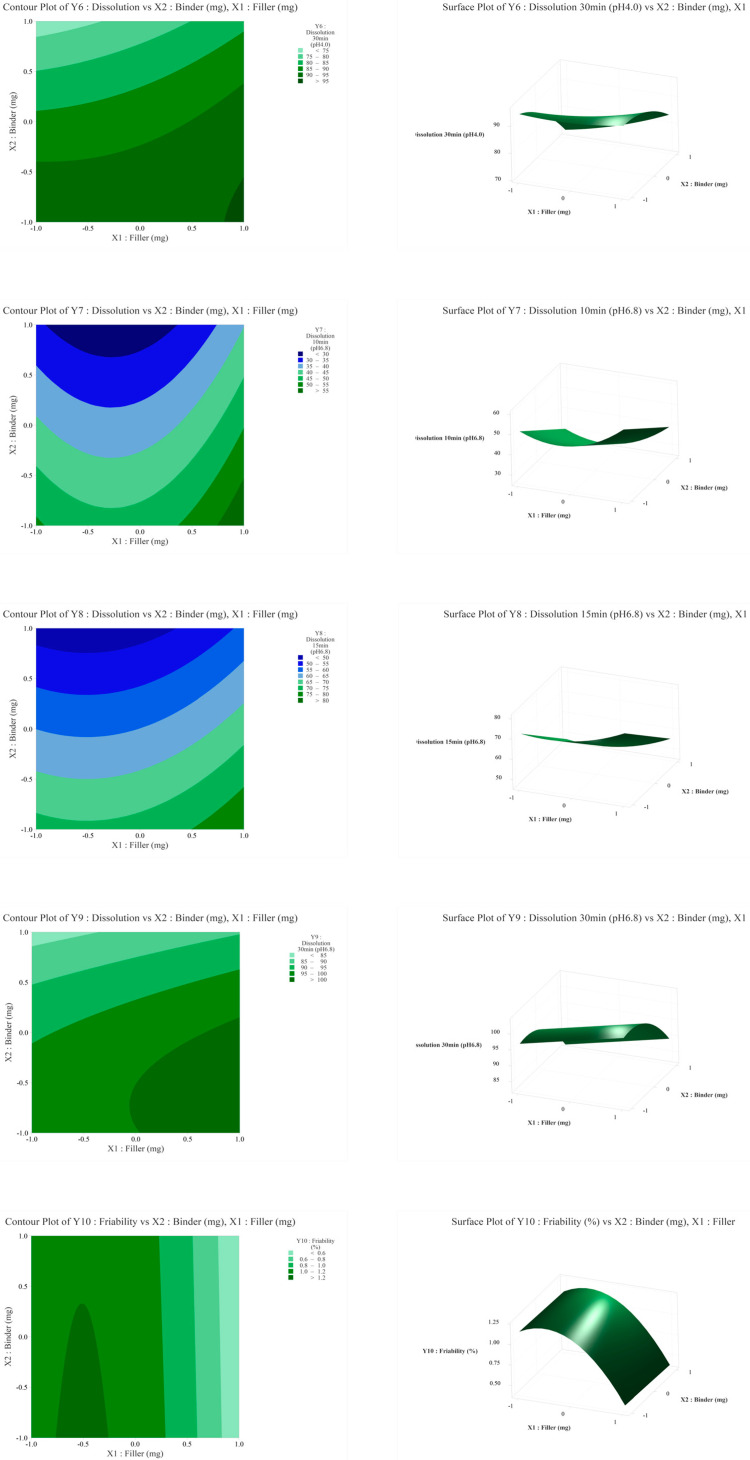

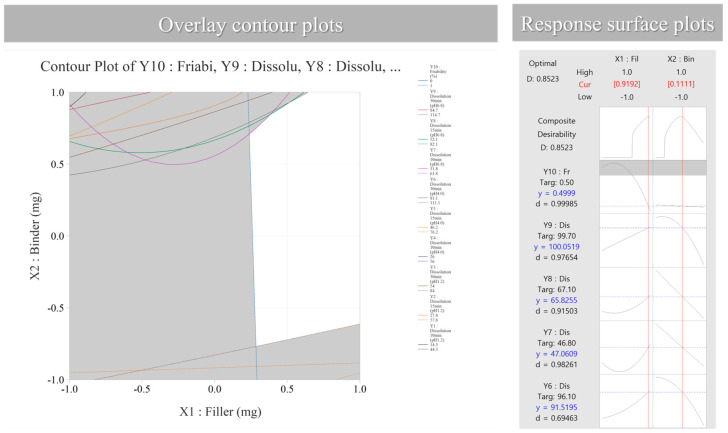
Visualization of semaglutide critical material attributes using pareto, residual, factorial, contour, overlay, and response surface plots.

These findings are supported by the Pareto analysis, residual diagnostics, and response surface modeling presented in Figure 6. ANOVA further confirmed that the linear terms of filler (X_1_) and binder (X_2_) significantly influenced the response (*p* = 0.006 and *p* < 0.001, respectively). In contrast, the quadratic term of filler (X_1_^2^) was not statistically significant (*p* = 0.269), while the interaction term (X_1_X_2_) showed a marginal effect (*p* = 0.054).

The statistical significance of model terms was evaluated using ANOVA with a significance threshold of *p* < 0.05, and the relative contribution of these factors was visualized using Pareto charts. Furthermore, response optimization was conducted using a desirability function approach. The desirability criteria were defined to target dissolution similarity to the reference product (*f*_2_ ≥ 50) while maintaining acceptable mechanical robustness of the tablets.

Response surface and contour plot analysis showed that setting the binder level at its central value enabled identification of an optimal design space that satisfied all CQA criteria across a relatively broad filler range.

Overall, this formulation optimization study identified binder level as a key CMA for ensuring semaglutide tablet quality, while filler functioned primarily as a robustness factor with minimal effect on performance. The establishment of a robust design space, in which filler served as a non-critical variable, demonstrated that the formulation could maintain consistent quality despite minor composition fluctuations. By empirically distinguishing critical from non-critical factors, this study validates the predictability and reliability of the applied QbD-guided approach. Specifically, the design space was established under conditions where the absorption enhancer content was fixed, highlighting the utility of a QbD-guided approach in managing formulation complexity introduced by functional excipients.

### 3.5. Characteristic Evaluation of Optimized Semaglutide Tablets

**Figure 7:**
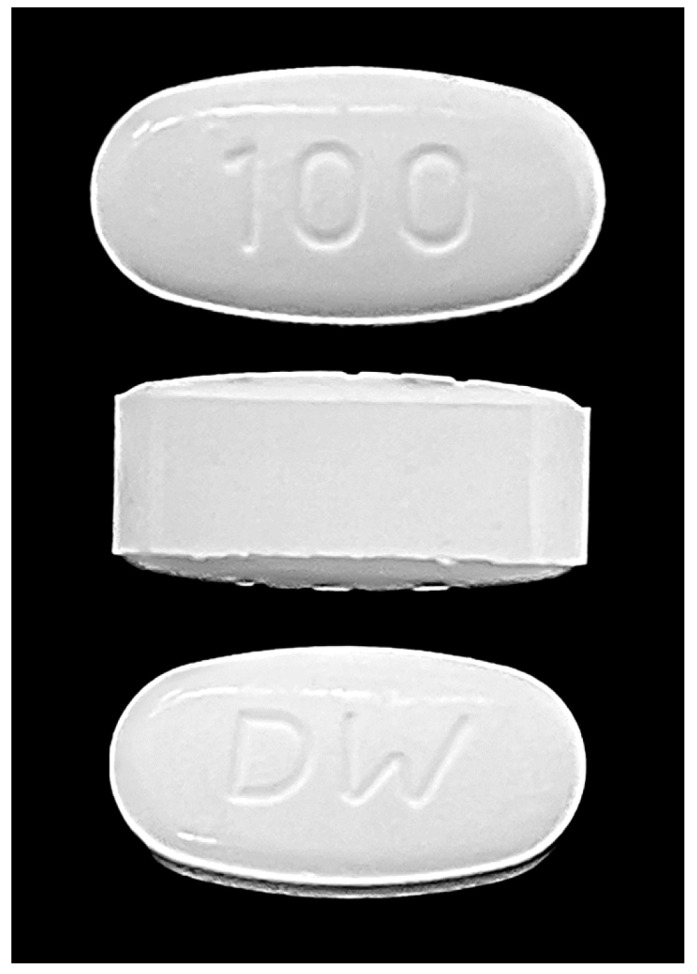
Representative photographs of the optimized semaglutide tablets developed in this study, showing the top, side, and bottom views of the tablets.

Optimized semaglutide tablets manufactured based on DoE-driven response optimization were evaluated for key quality attributes using the methods described in Section 2.7. Table 8 depicts that all tablets met the predefined acceptance criteria for mechanical strength and content uniformity. Representative photographs of the optimized semaglutide tablets, including the top, side, and bottom views, are presented in Figure 7.

Tablet hardness, measured using a digital hardness tester, ranged from 7 to 9 kp, indicating adequate mechanical strength for handling, distribution, and storage. Friability was 0.57%, meeting the ≤1.0% acceptance criterion and demonstrating sufficient resistance to mechanical stress. Additionally, disintegration testing conducted in purified water at 37 ± 0.5 °C showed complete disintegration of all tablets within 14 min and 14 s, satisfying the specified acceptance limit and indicating prompt drug release.

HPLC assay results showed a semaglutide content of 98.3%, falling within the acceptable range of 90–110%. Furthermore, content uniformity testing performed in accordance with USP <905> confirmed that all individual tablets met the acceptance criteria. Collectively, these findings indicate that the optimized formulation achieved satisfactory tablet quality and manufacturing reliability. However, one potential limitation of the present study relates to the use of dry granulation as the manufacturing process. Although this approach was selected to ensure peptide stability and manufacturing feasibility, the spatial distribution between semaglutide and the absorption enhancer within the tablet matrix may influence enhancer-mediated transport efficiency.

To address this limitation, future studies may explore alternative formulation strategies aimed at improving API–enhancer proximity within the solid matrix, such as approaches that enable closer co-localization of the API and the absorption enhancer. In addition, more detailed spatial characterization may be conducted using imaging-based techniques (e.g., SEM mapping) or solid-state analytical methods (e.g., XRPD or spectroscopic analysis) to further elucidate API–enhancer interactions and their spatial distribution within the dosage form.

### 3.6. In Vitro Dissolution Comparison with Reference Product

The in vitro dissolution behavior of the optimized semaglutide tablet was evaluated under biorelevant pH conditions (1.2, 4.0, and 6.8) and compared with that of the reference product, Rybelsus^®^. Dissolution testing was conducted using media containing 0.75% Brij^®^ 35 at pH 1.2 and 4.0, while at pH 6.8, testing was conducted under standard comparative dissolution conditions without surfactant, in accordance with regulatory guidelines.

**Figure 8:**
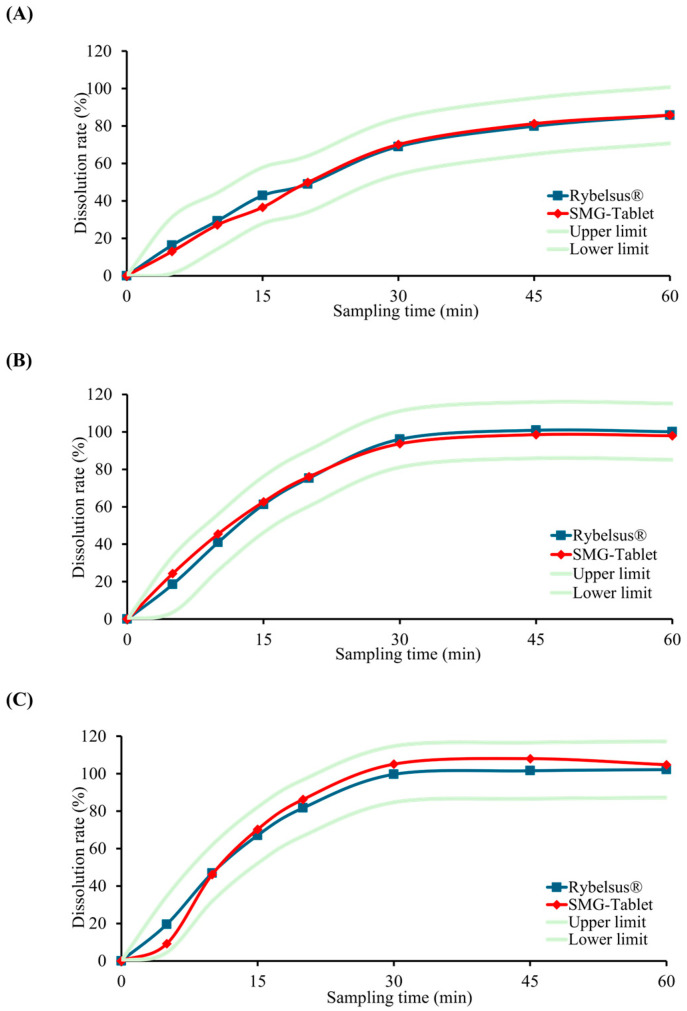
Comparative dissolution profiles of the optimized semaglutide tablet and reference product (Rybelsus^®^) in various biorelevant media: (**A**) pH 1.2 with 0.75% Brij^®^ 35, (**B**) pH 4.0 with 0.75% Brij^®^ 35, and (**C**) pH 6.8 (*n* = 12). The shaded area represents the ±15% acceptance range relative to the mean dissolution of the reference product, in accordance with regulatory guidelines for in vitro equivalence assessment.

The optimized formulation demonstrated consistent and predictable release profiles across all pH conditions, closely matching those of the reference product. Calculated f_2_ values were 74.4 at pH 1.2, 74.7 at pH 4.0, and 71.3 at pH 6.8, all exceeding the regulatory threshold of 50, confirming statistical similarity with the reference product. Comparative dissolution profiles of the optimized formulation and the reference product across biorelevant pH conditions are presented in Figure 8.

These findings indicate that the developed formulation satisfies all predefined quality criteria and achieves a dissolution profile highly similar to that of the commercial product. Accordingly, the optimized semaglutide tablet fulfilled the established QTPP requirements and demonstrated in vitro dissolution similarity to the reference product, suggesting a potential for comparable in vivo performance; however, this remains hypothetical and requires further clinical validation. Overall, these findings underscore the feasibility of the formulation as a potential clinical oral semaglutide dosage form.

## 4. Conclusions

This study systematically evaluated a C10-based oral semaglutide formulation using a QbD-driven approach and demonstrated the utility of this framework for formulation optimization.

A comprehensive evaluation of the physicochemical properties of semaglutide established its solubility profile across physiological pH conditions, while excipient compatibility screening detected specific excipients with the potential to compromise stability. These data provided a rational foundation for formulation design. Through QTPP definition, CQA selection, and RA using PHA and FMEA, binder level was identified as a key CMA, while filler primarily functioned as a robustness factor. Subsequent DoE-based optimization using a CCD confirmed binder content as the primary determinant influencing tablet mechanical properties and dissolution performance. The optimized formulation satisfied all predefined quality criteria—including hardness, friability, disintegration, and content uniformity—while maintaining mechanical robustness and dosage consistency, supporting the reproducibility and reliability of the QbD-based development approach. In vitro dissolution studies further demonstrated in vitro dissolution similarity with the reference product, with f_2_ values > 50 across all biorelevant pH conditions.

Taken together, these results provide formulation-level insight into the design and optimization of C10-containing oral semaglutide systems and support the utility of a QbD-guided strategy for systematically evaluating mechanistically distinct intestinal permeation enhancer-based formulations. Further in vivo bioavailability studies, clinical investigations, and stability-indicating impurity profiling will be needed to more fully evaluate the translational relevance and pharmaceutical characterization of this formulation. Collectively, such follow-up studies may further clarify the applicability of this formulation strategy in oral peptide delivery and provide a useful foundation for future academic research on peptide-based oral delivery systems.

## Figures and Tables

**Table 1:** QTPP.

QTPP	Target	Risk	Justification
Indication	Type 2 diabetes	Yes	This formulation functions as a GLP-1 receptor agonist and is intended to enhance glycemic control in patients with type 2 diabetes. Clearly defining the therapeutic indication ensures alignment between clinical application and pharmacological activity, thereby reducing the potential for inappropriate or off-label use.
Dosage form	Immediate- release uncoated tablet	Yes	This dosage form is designed to rapidly disintegrate in the stomach and release the drug for timely transit to the upper intestine, where C10 enhances absorption. Since C10 primarily acts in the drug absorption in the small intestine, prompt gastric disintegration is critical to ensure effective exposure at the intended absorption site. Any delay in disintegration or prolonged release could compromise absorption efficiency and reduce overall bioavailability.
Route of administration	Oral administration	No	Oral delivery is one of the most preferred administration routes owing to improved patient convenience and adherence compared with parenteral injectable formulations.
Dosage strength	14 mg once daily	No	A fixed dosage strength equivalent to the reference product (Rybelsus^®^ 14 mg) is selected to support therapeutic equivalence and ensure consistent systemic exposure.
Appearance	White to light yellow, oblong tablets	No	Tablet shape and color are important for product recognition; however, they do not directly affect safety or therapeutic performance, and thus represent a low-risk attribute.
Identification	Equivalent requirement for peak dwell time	No	Identification testing does not directly affect product safety or therapeutic efficacy, and it does not pose a critical risk provided that the purity of the API is consistently ensured.
Assay	90.0–110.0% (semaglutide)	Yes	Peptides are high-potency, low-dose therapeutics; therefore, even small deviations in assay content can influence therapeutic performance. Excessive drug content may increase the risk of adverse effects, whereas insufficient content may lead to inadequate pharmacological response. Consequently, precise control of assay content is essential to ensure consistent product quality and reliable therapeutic outcomes.
Content uniformity	Complies with USP <905> (AV ≤ 15)	Yes	Content uniformity is essential for dosing accuracy and patient safety, as non-uniform distribution can lead to dose variability, thereby affecting safety and therapeutic efficacy. In peptide formulations containing absorption enhancers, achieving uniform distribution is particularly challenging. Therefore, rigorous control of blending uniformity and compression parameters during dry granulation is essential to ensure consistent product quality.
Dissolution	Dissolution performance is targeted to be comparable to the reference product when tested under USP Apparatus II conditions across three dissolution media (pH 1.2, 4.0, and 6.8). Final comparative dissolution is evaluated using similarity criteria, with an f_2_ value ≥ 50, to demonstrate in vitro equivalence.	Yes	While this formulation was developed as a generic product, achieving dissolution behavior comparable to the reference product was established as a key quality objective. Therefore, comparative dissolution testing was conducted in three dissolution media—pH 1.2, 4.0, and 6.8—under USP Apparatus II. Dissolution similarity to the reference product—defined by f_2_ ≥ 50—was set as the acceptance criterion to support the demonstration of in vitro equivalence and ensure consistent dissolution performance of the final product.
Disintegration	Rapid disintegration suitable for immediate-release tablet	Yes	Rapid disintegration is required to enable timely drug release and ensure drug availability for intestinal absorption in the presence of C10.
Tablet mechanical integrity	Adequate hardness with low friability	Yes	Mechanical robustness is necessary to maintain tablet integrity during manufacturing, packaging, and handling.

Abbreviations: C10, sodium caprate; QTPP, Quality Target Product Profile; API, active pharmaceutical ingredient; USP, United States Pharmacopeia; AV, acceptance value; f_2_, similarity factor. Note: red indicates high risk, yellow indicates medium risk, and green indicates low risk.

**Table 2:** CQAs.

QAs of the Immediate -Release Tablet	Objective	CQA	Justification
Appearance	The tablet should have a convenient shape, color and size to support patient compliance and facilitate ease of administration.	No	Tablet color and appearance may influence patient adherence; however, they do not directly influence drug safety or therapeutic efficacy and are therefore not considered CQAs.
Identification	APIs should be identifiable based on their equivalent peak retention time	No	Identification testing is essential for confirming product safety and therapeutic efficacy; however, it is readily controlled and monitored during routine quality control. Thus, formulation and process parameters have a limited influence on this attribute.
Assay	90.0–110.0% (semaglutide)	Yes	Variations in drug content can influence product safety and therapeutic performance. Because assay variability directly affects dose accuracy and overall product quality, the assay is designated as a CQA and requires strict control during formulation and process development.
Content uniformity	90.0–110.0% of label claim; AV ≤ 15.0%; RSD ≤ 5.0%	Yes	Variability in content uniformity can directly affect product safety and therapeutic performance. Since this attribute may be affected by formulation design and manufacturing parameters, it must be systematically monitored and controlled throughout formulation development and process optimization.
Dissolution	Dissolution profiles should be comparable to the reference product using USP <711> Apparatus II (paddle, 50 rpm), across three media (pH 1.2, 4.0, and 6.8). Similarity is assessed using the f_2_ criterion (f_2_ ≥ 50) for each medium.	Yes	Since this formulation was developed as a generic product, dissolution similarity with the reference product is a CQA for demonstrating in vitro equivalence. Comparative dissolution testing across multiple pH conditions (1.2, 4.0, and 6.8) ensures consistent drug release under physiologically relevant conditions. Dissolution similarity, defined as an f_2_ value ≥ 50 in each medium, was established as a key criterion to meet regulatory expectations and confirm consistent product quality.

Abbreviations: CQAs, critical quality attributes; QA, quality attributes; USP, United States Pharmacopeia; AV, acceptance value; RSD, relative standard deviation; f_2_, similarity factor. Note: red indicates high risk, yellow indicates medium risk, and green indicates low risk.

**Table 3:** Composition of the optimized semaglutide monolayer tablet formulation.

Ingredient	Function	Amount (mg/Tablet)
Semaglutide	Active pharmaceutical ingredient (API)	14
Lactose monohydrate	Filler	300
Sodium caprate (C10)	Absorption enhancer	300
Croscarmellose sodium	Disintegrant	17
Copovidone	Binder	13
Magnesium stearate	Lubricant	8
Total weight		652 mg

**Table 4:** Results of the compatibility test between semaglutide and excipients.

Sample (1:1, *w*/*w*)	Initial (%)	RT		AC	
		2 w	4 w	2 w	4 w
Sema	99.5	97.2	97.7	98.7	97.5
Sema:MCC	99.9	99.9	101.0	98.7	98.9
Sema:Lactose	98.9	97.9	99.2	97.1	97.7
Sema:L-HPC	98.8	96.9	101.1	99.6	98.6
Sema:SSG	99.5	103.0	101.2	101.3	100.1
Sema:Crospovidone	99.0	101.3	101.5	98.8	97.9
Sema:CCS	99.4	101.5	102.6	100.5	101.6
Sema:Mg.S	97.3	98.7	97.7	98.4	96.4
Sema:Aerosil	97.7	98.0	98.7	97.1	96.9
Sema:Talc	96.6	98.8	96.7	99.7	99.3
Sema:HPMC	98.6	97.4	101.4	97.8	97.5
Sema:Povidone K90	98.8	99.8	101.0	98.4	97.9
Sema:Copovidone VA64 fine	100.1	102.1	99.7	98.1	98.9
Sema:PEG	98.1	97.4	99.9	96.9	97.5
Sema:SLS	90.9	97.1	0.2	91.8	0.2
Sema:Sodium hydrogen carbonate	98.3	96.1	98.8	96.8	97.6
Sema:Sodium carbonate	99.5	98.8	100.3	99.6	101.5
Sema:Mg.C	96.8	98.3	96.9	96.6	97.5
Sema:Salicylic acid	0.0	0.0	0.0	0.0	0.0
Sema:SNAC	101.0	98.4	98.6	98.3	98.7
Sema:C10	99.3	99.5	100.5	99.8	99.3

Abbreviations: Sema, semaglutide; MCC, microcrystalline cellulose; L-HPC, L-hydroxypropyl cellulose; SSG, sodium starch glycolate; CCS, croscarmellose sodium; Mg.S, magnesium stearate; HPMC, hypromellose; PEG 80, Polyethylene glycol 80; SLS, sodium lauryl sulfate; Mg.C, magnesium carbonate; SNAC, sodium *N*-[8-(2-hydroxybenzoyl)amino]caprylate; C10, sodium caprate.

**Table 5:** Pharmacokinetic parameters of semaglutide following oral administration in Sprague–Dawley rats (*n* = 5).

PK Parameter	PK Parameter (Mean ± SD)			
	Group 1	Group 2	Group 3	Group 4
	Semaglutide	Semaglutide + C10 100 mg/kg	Semaglutide + C10 200 mg/kg	Semaglutide + SNAC 200 mg/kg
t_max_ (h)	3.0 ± 2.7	1.3 ± 0.9	1.8 ±1.4	1.5 ± 1.5
C_max_ (ng/mL)	9.9 ± 13.4	23.3 ± 39.1	30.4 ± 53.2	43.5 ± 44.4
AUC_last_ (ng·h/mL)	58.9 ± 76.4	230.5 ± 431.6	346.9 ± 719.8	503.1 ± 586.3
AUC_inf_ (ng·h/mL)	211.6	482.0 ± 652.8	630.5 ± 981.9	939.6 ± 618.3
t_1/2_ (h)	4.4	4.7 ± 2.3	4.1 ± 2.2	7.2 ± 0.7

Abbreviations: SD, Standard deviation; PK, pharmacokinetic; t_max_, time to maximum plasma concentration; C_max_, maximum plasma concentration; AUC_last_, area under the plasma concentration–time curve to last measurable concentration; AUC_inf_, area under the concentration–time curve extrapolated to infinity; t_1/2_, elimination half-life.

**Table 6:** Results of risk assessment. (**A**) PHA and (**B**) FMEA of MAs.

(**A**)								
CQA	Semaglutide	Absorption Enhancer	Filler	Binder	Disintegrant		Lubricant	
Identification	Low	Low	Low	Low	Low		Low	
Assay	Low	Low	Low	Low	Low		Low	
Uniformity	Low	Medium	Low	Low	Low		Low	
Dissolution	Medium	High	High	High	Medium		Low	
(**B**)								
Unit Operation	CMAs	Failure Mode (Critical Event)	Justification of Failure Mode		P	S	D	RPN
API property	Solubility	Different salt	It may influence tablet release behavior; however, its impact is considered relatively limited under fixed API form and formulation conditions applied in this study.		2	2	1	4
Filler	Proportion of filler	Higher than optimum	Excessive lactose increases water-soluble content, which can accelerate the dissolution rate. This may deviate from the target dissolution profile, potentially affecting drug efficacy and bioavailability.		3	4	4	48
		Lower than optimum	Insufficient lactose content increases the relative absorption enhancer, leading to a more hydrophobic microenvironment. This may delay dissolution.		3	3	5	45
Binder	Proportion of binder	Higher than optimum	Excess binder increases hardness, which can delay disintegration/ dissolution, ultimately reducing dissolution rate and delaying drug release, potentially affecting in vitro performance relative to the reference product.		4	3	4	48
		Lower than optimum	Insufficient binder reduces interparticle bonding strength, making the tablet more prone to cracking or breaking. This may affect disintegration and dissolution reproducibility, resulting in deviation from the target dissolution profile and reduced mechanical integrity of the tablet.		4	4	3	48
Disintegrant	Proportion of disintegrant	Higher than optimum	Too fast drug release may result in a deviate from reference dissolution and a decrease in hardness, which may compromise overall product quality and dissolution similarity.		4	3	2	24
		Lower than optimum	Delayed disintegration may prevent the target dissolution profile, potentially resulting in deviation from the target dissolution behavior.		3	3	3	27
Absorption enhancer	Proportion of absorption enhancer	Higher than optimum	Excess levels may alter the local microenvironment, resulting in a deviation from the intended absorption profile, potentially affecting absorption performance.		2	4	2	16
		Lower than optimum	Insufficient levels may reduce intestinal drug permeability, potentially resulting in inadequate absorption.		3	5	1	15
Lubricant	Proportion of lubricant	Lower than optimum	Picking and sticking during compression may damage the quality.		3	2	3	18

Abbreviations: CQA, critical quality attribute; CMAs, critical material attributes; P, probability; S, severity; D, detectability; RPN, risk priority number; PHA, Preliminary Hazard Analysis; FMEA, Failure Mode Effects Analysis; MAs, material attributes; API, active pharmaceutical ingredient. Note: red indicates high risk, yellow indicates medium risk, and green indicates low risk.

**Table 7:** CMAs affecting CQAs of semaglutide (coded values).

Run	^a^CMA		^b^CQAs									
	X1	X2	Y1	Y2	Y3	Y4	Y5	Y6	Y7	Y8	Y9	Y10
1	−1	−1	46.9	64.7	88.8	52.7	70.3	94.3	55.0	75.5	99.7	1.16
2	+1	−1	42.8	58.1	87.5	52.1	72.3	96.1	57.9	79.1	100.0	0.35
3	−1	+1	15.4	26.6	47.8	29.1	40.7	72.6	32.1	46.9	82.3	0.77
4	+1	+1	23.2	28.1	55.2	36.8	51.3	84.2	45.1	61.9	89.6	0.45
5	−1	0	22.1	31.1	54.8	39.0	55.0	85.7	35.7	57.5	89.0	1.44
6	+1	0	33.8	42.4	76.2	48.8	68.7	91.8	46.3	63.3	101.0	0.46
7	0	−1	41.3	58.1	87.0	53.0	75.0	92.7	49.0	73.0	99.5	0.97
8	0	+1	19.0	28.1	53.5	35.8	50.4	76.4	24.8	46.9	86.7	1.16
9	0	0	26.3	37.3	69.0	44.8	58.1	89.1	37.6	59.7	98.2	1.2
10	0	0	27.1	36.5	68.4	45.2	57.6	88.4	38.1	61.1	98.8	1.18
11	0	0	25.6	37.0	68.9	43.0	56.9	88.0	38.4	61.3	98.7	1.06
12	0	0	25.1	35.1	67.7	46.1	58.3	89.3	36.5	58.9	97.8	1.15
13	0	0	26.8	35.9	68.1	42.2	55.6	87.6	37.1	59.6	100.0	1.09

Abbreviations: ^a^CMA, critical material attribute; ^b^CQAs, critical quality attributes. Note: X1 and X2 represent filler and binder levels, respectively, evaluated using coded values (−1, 0, +1). The actual quantitative ranges corresponding to the coded levels were 50–300 mg for the filler (lactose) and 0–46 mg for the binder (copovidone). Y1–Y3 = Dissolution at pH 1.2 (10, 15, 30 min); Y4–Y6 = Dissolution at pH 4.0 (10, 15, 30 min); Y7–Y9 = Dissolution at pH 6.8 (10, 15, 30 min); Y10 = Friability (%).

**Table 8:** CQAs of optimized semaglutide tablets.

Quality Attribute	Method/Condition	Acceptance Criteria	Result (Mean ± SD)	*n*
Hardness (kp)	Digital hardness tester	7–9 kp	7.8 ± 0.4	6
Friability (%)	USP <1216>	≤1.0%	0.57	10
Disintegration time	Purified water, 37 ± 0.5 °C	≤20 min	14.2 ± 1.1 min	6
Assay (%)	HPLC	90–110%	98.3 ± 1.2	6
Content uniformity	USP <905>	AV ≤ 15.0	Pass (AV = 6.8)	10

Abbreviations: kp, kilopond; USP, United States Pharmacopeia; HPLC, high-performance liquid chromatography; AV, acceptance value; SD, standard deviation; CQAs, Critical Quality Attributes.

## Data Availability

The original contributions presented in this study are included in the article. Further inquiries can be directed to the corresponding author.

## Abbreviations

API	Active pharmaceutical ingredient
AV	Acceptance value
CCD	Central composite design
CMA	Critical material attribute
CQA	Critical quality attribute
C10	Sodium caprate
DoE	Design of experiments
FMEA	Failure mode and effects analysis
GLP-1	Glucagon-like peptide-1
GLP-1 RA	Glucagon-like peptide-1 receptor agonist
HPLC	High-performance liquid chromatography
HPMC	Hypromellose
L-HPC	L-hydroxypropyl cellulose
MAs	Material attributes
MCC	Microcrystalline cellulose
Mg.C	Magnesium carbonate
Mg.S	Magnesium stearate
PHA	Preliminary hazard analysis
PEG 80	Polyethylene glycol 80
QbD	Quality by design
QTPP	Quality target product profile
RPN	Risk priority number
RSD	Relative standard deviation
CCS	Croscarmellose sodium
Sema	Semaglutide
SLS	Sodium lauryl sulfate
SNAC	Sodium *N*-[8-(2-hydroxybenzoyl)amino]caprylate
SSG	Sodium starch glycolate
SD	Standard deviation
USP	United States Pharmacopeia
f_2_	Similarity factor
kp	Kilopond
